# In ovo injection of betaine alleviates corticosterone-induced fatty liver in chickens through epigenetic modifications

**DOI:** 10.1038/srep40251

**Published:** 2017-01-06

**Authors:** Yun Hu, Qinwei Sun, Jie Liu, Yimin Jia, Demin Cai, Abdulrahman A. Idriss, Nagmeldin A. Omer, Ruqian Zhao

**Affiliations:** 1 Key Laboratory of Animal Physiology & Biochemistry, Nanjing Agricultural University, Nanjing 210095, P. R. China; 2 Jiangsu Collaborative Innovation Centre of Meat Production and Processing, Quality and Safety Control, Nanjing 210095, P. R. China

## Abstract

Betaine alleviates high-fat diet-induced fatty liver and prenatal betaine programs offspring hepatic lipid metabolism. Excessive corticosterone (CORT) exposure causes fatty liver in chickens, yet it remains unknown whether and how prenatal betaine modulates the susceptibility of CORT-induced fatty liver later in life. In this study, fertilized eggs were injected with saline or betaine before incubation, and the hatchlings were raised at 8 weeks of age followed by 7 days of subcutaneous CORT injection. CORT-induced fatty liver was less severe in betaine-treated chickens, with significantly reduced oil-red staining and hepatic triglyceride content (*P* < 0.05). The protective effect of prenatal betaine was associated with significantly up-regulated expression of *PPARα* and *CPT1α*, as well as mitochondrial DNA (mtDNA)-encoded genes (*P* < 0.05). Moreover, betaine rescued CORT-induced alterations in methionine cycle genes, which coincided with modifications of CpG methylation on *CPT1α* gene promoter and mtDNA D-loop regions. Furthermore, the elevation of hepatic GR protein content after CORT treatment was significantly reduced (*P* < 0.05), while the reduction of GR binding to the control region of affected genes was significantly increased (*P* < 0.05), in betaine-treated chickens. These results indicate that *in ovo* betaine injection protects the juvenile chickens from CORT-induced fatty liver.

Non-alcoholic fatty liver disease (NAFLD) is the hepatic manifestation of metabolic syndrome, which may lead to severe liver failure^1^,^2^,^3^. Glucocorticoid (GC) excess is a common feature of NAFLD^4^ that is associated with chronically elevated GC level in human clinical studies^5^. In chickens, corticosterone (CORT) is the main active form of GC and also a reliable indicator of stress. Exogenous CORT administration causes abnormal accumulation of fat in the liver^6^,^7^, which is manifested with mitochondrial dysfunction, lipid peroxidation and inflammation, thereby increasing the susceptibility of liver to more severe damages^6^,^8^.

Lipid homeostasis in the liver is maintained through highly coordinated and strictly regulated biological processes including lipid biosynthesis, lipolysis, as well as fatty acids transport and β-oxidation^9^,^10^,^11^. Mitochondrial β-oxidation plays a key role in the pathogenesis of NAFLD^12^,^13^. Fatty acids β-oxidation in the liver is regulated by carnitine palmitoyl transferase 1 (CPT-1) that catalyzes the rate-limiting step of fatty acid oxidation^2^,^10^, and the key transcription factor peroxiisome proliferator-activated receptor alpha (PPARα) that activates mitochondrial β-oxidation and oxidative phosphorylation (OXPHOS)^14^. Hepatic mitochondrial β-oxidation and OXPHOS were regulated by GCs, primarily through binding and activating its intracellular glucocorticoid receptor (GR)^15^,^16^. GR, a transcription factor belonging to the superfamily of nuclear receptors, can also translocate to mitochondria to regulate the replication and transcription of mitochondrial DNA (mtDNA) in mammals^17^ and HepG2 cells^18^. However, it remains unclear whether GR is involved in the mitochondrial dysfunction in CORT-induced fatty liver in chickens.

Betaine, a methyl donor, is an important component of the methionine cycle which is essential for the epigenetic gene regulation through DNA methylation and histone modifications^19^,^20^,^21^. It is well-documented that betaine can promote growth and improve carcass characteristics and meat quality in livestock animals^22^,^23^ and broiler chickens^24^,^25^. Moreover, betaine deficiency is associated with NAFLD severity^26^,^27^. Betaine has been demonstrated to be effective in treating NAFLD in mammals^27^,^28^,^29^,^30^ and betaine supplementation can alleviate high-fat diet-induced hepatic lipid accumulation in rats via enhancing hepatic lipid export and fatty acid oxidation^30^,^31^. DNA methylation and histone modifications are involved in regulating hepatic lipid metabolism in offspring piglets born to betaine-treated sows^32^. Also, *in ovo* injection of betaine affects hepatic cholesterol metabolism in newly hatched chicks through epigenetic gene regulation^33^. Nevertheless, it remains unknown whether betaine *in ovo* may alleviate CORT-induced hepatic fat deposition through epigenetic regulation of mitochondrial β-oxidation and OXPHOS genes in chickens later in life.

Betaine supplementation was reported to significantly decrease the circulating cortisol level in athletes in response to acute bouts of exercise^34^. Recently, it is reported that gestational dietary betaine supplementation decreased plasma cortisol level in neonatal pig offspring^32^. Hence questions arise whether plasma CORT is altered by prenatal betaine exposure, and how this is associated with GR-mediated mitochondrial dysfunction in CORT-induced fatty liver in chickens.

Therefore, the objectives of the present study were to investigate: 1) whether *in ovo* betaine injection could alleviate CORT-induced fatty liver in juvenile chickens; 2) how such effects, if any, are related to epigenetic regulation of mitochondrial β-oxidation and OXPHOS genes; 3) whether plasma CORT level and hepatic GR binding to the promoter of affected genes are also involved in the mechanisms.

## Results

### Body weight and feed intake

**Figure 1:**
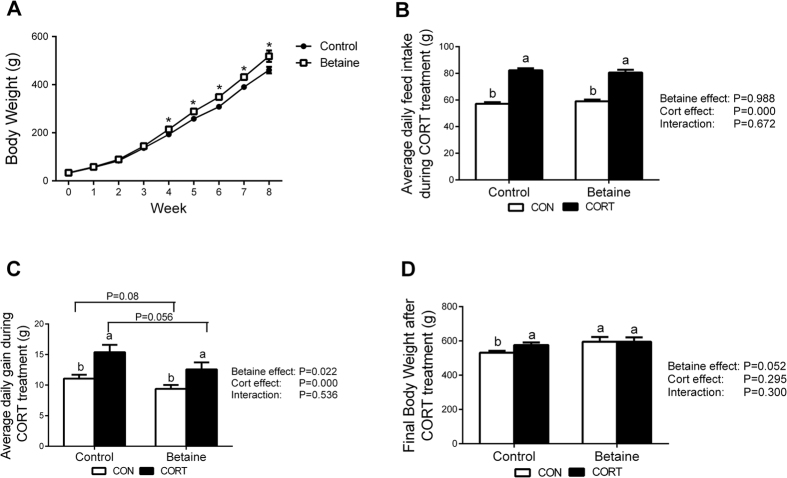
Chicken body weight and growth performance with *in ovo* injection of betaine and subsequent 7 days of CORT challenge. (**A**) Chicken body weight; **(B**) Average daily feed intake; (**C**) Average daily gain; (**D**) Final body weight during 7 days of CORT treatment. C = Control; B = Betaine; CON = vehicle; CORT = Corticosterone; Values are means ± SEM, ^*^P < 0.05 VS Betaine group, means without a common letter differ, *P* < 0.05 (n = 8).

Betaine-treated chickens demonstrated significantly higher (*P* < 0.05) body weight from 4th to 8th week post hatching (Fig. 1A). Betaine had no effect on the average daily feed intake (Fig. 1B), but slightly yet significantly decreased (*P* < 0.05) the daily gain (Fig. 1C). However, CORT challenge significantly increased (*P* < 0.05) the average daily feed intake (Fig. 1B) and daily gain (Fig. 1C) in both Control and Betaine groups. At the end of CORT challenge, the final body weight (Fig. 1D) in Control chickens was significantly higher (*P* < 0.05), but that in Betaine group remained unaltered.

### Phenotypic characterization of fatty liver syndrome

**Figure 2:**
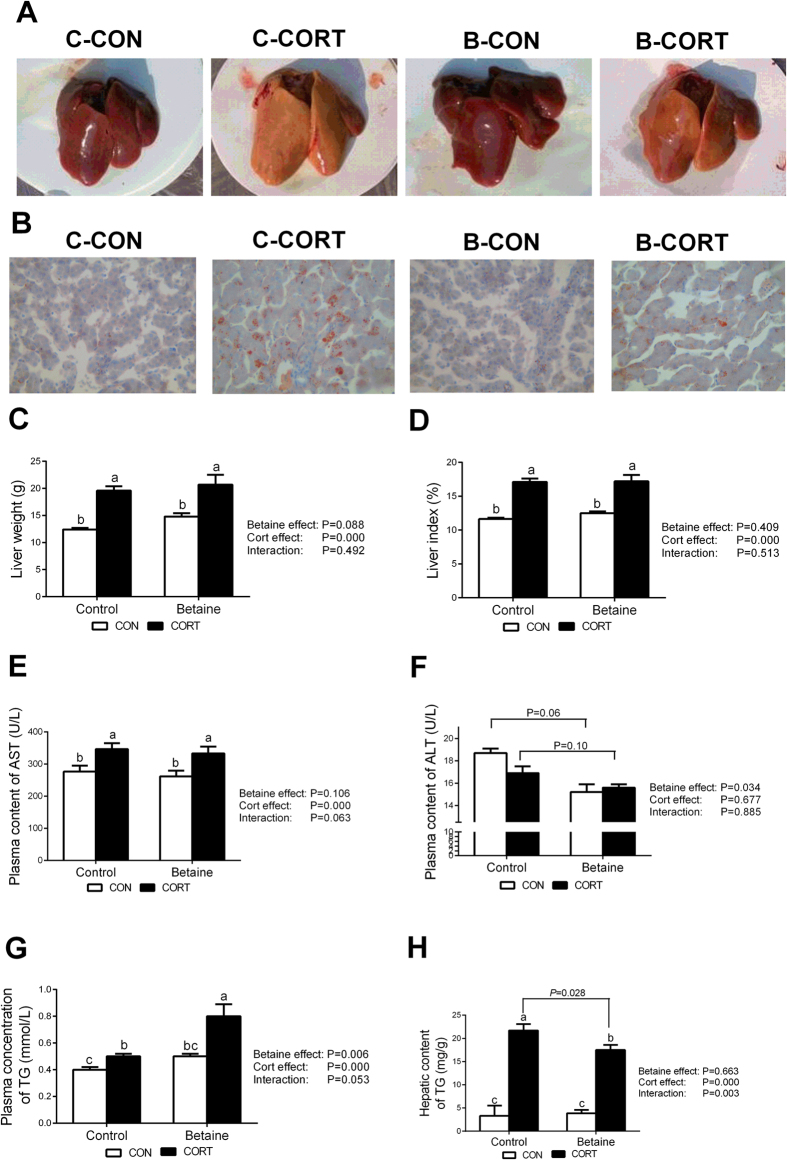
Phenotypic characterization of fatty liver syndrome. (**A**) Phenotypic character of liver; (**B**) Histological sections stained with oil-red; (**C**) Liver weight; (**D**) Liver index; (**E**) Plasma content of AST; (**F**) Plasma content of ALT; (**G**) Plasma concentration of TG; (**H**) Hepatic content of TG. C = Control; B = Betaine; CON = vehicle; CORT = Corticosterone; The liver index is the ratio of liver weight relative to the body weight. Values are means ± SEM, means without a common letter differ, *P* < 0.05 (n = 8).

Betaine injection *in ovo* did not change the liver color in chickens not subjected to CORT challenge. Chronic CORT exposure changed the liver color from red to yellow in Control group (Fig. 2A), and prenatal betaine exposure significantly alleviated such pathological alteration (Fig. 2A). The same phenomenon was seen in histological sections stained with oil-red to show hepatic lipid accumulation (Fig. 2B). *In ovo* injection of betaine had no effect on liver weight (Fig. 2C) or liver index (Fig. 2D). Chronic CORT administration significantly increased (*P* < 0.05) the liver weight (Fig. 2C) and the liver index (Fig. 2D), both in Control and Betaine groups. Plasma activity of aspertate aminotransferase (AST) was also significantly (*P* < 0.05) elevated in response to CORT challenge in both Control and Betaine groups (Fig. 2E), which indicates liver injury and metabolic adaptation^35^. In contrast, alanine aminotransferase (ALT), another plasma index for liver functionality, was not affected by CORT, yet significantly decreased (*P* < 0.05) in betaine groups (Fig. 2F). CORT had significant effects on triglyceride (TG) content in plasma (Fig. 2G) and liver (Fig. 2H). Post hoc analysis revealed significant elevation (*P* < 0.05) in plasma concentration (Fig. 2G) and hepatic content (Fig. 2H) of total TG under CORT challenge in both Control and Betaine groups. However, prenatal betaine exposure significantly augmented CORT-induced elevation in plasma TG (*P* < 0.05), while reduced CORT-induced hepatic TG deposition (*P* < 0.05).

### Hepatic expression of methionine cycle and methyl transfer genes

**Figure 3:**
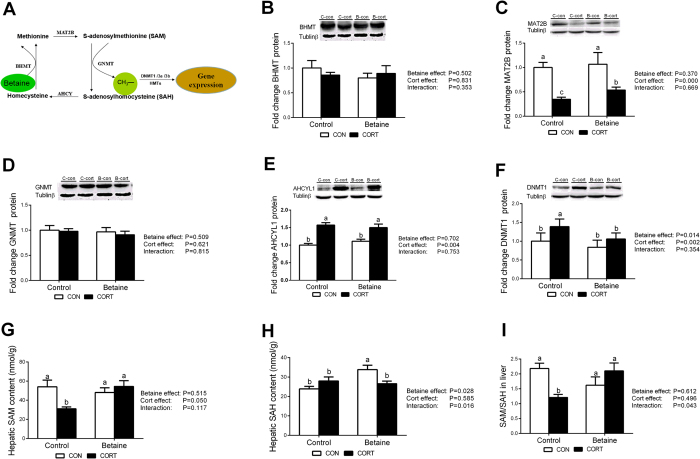
Hepatic protein content of enzymes and metabolites involved in methionine cycle and methyl transfer in chickens. (**A**) Schematic diagram of methionine cycle; (**B**) Protein expression of BHMT; (**C**) Protein expression of MAT2B; (**D**) Protein expression of GNMT; (**E**) Protein expression of AHCYL1; (**F**) Protein expression of DNMT1; (**G**) Hepatic SAM content; (**H**) Hepatic SAH content; (**I**) The ratio of SAM/SAH in liver. C = Control; B = Betaine; CON = vehicle; CORT = Corticosterone; Values are means ± SEM, means without a common letter differ, *P* < 0.05 (n = 8).

The schematic diagram of methionine cycle is shown in Fig. 3A. Among the 4 enzymes involved in methionine cycle, MAT2B was not affected by betaine treatment, but significantly decreased (*P* < 0.05) after CORT challenge both in Control and Betaine groups (Fig. 3C). CORT-induced MAT2B down-regulation was partly alleviated (*P* < 0.05) in chickens received *in ovo* betaine injection. AHCYL1 was not affected by betaine treatment, but significantly increased (*P* < 0.05) after CORT treatment (Fig. 3E). Betaine homocysteine methyltransferase (BHMT) (Fig. 3B) and glycine N-methyltransferase (GNMT) (Fig. 3D) were not affected by either prenatal betaine injection or postnatal CORT challenge. Significant effects (P < 0.05) of both betaine and CORT were detected for DNA methyltransferase 1 (DNMT1) (Fig. 3F), yet post hoc analysis revealed significant increase (P < 0.05) in DNMT1 protein content only in Control groups, but not in Betaine groups (Fig. 3F), in response to CORT challenge. I*n ovo* betaine administration did not affect s-adenosyl methionine (SAM) (Fig. 3G), but increased significantly (*P* < 0.05) S-adenosyl homocysteine (SAH) (Fig. 3H) content in the liver under basal condition. In contrast, significant CORT effect was observed for SAM (*P* < 0.05), but not SAH. A Betaine and CORT interaction was observed for SAM/SAH ratio, which was significantly decreased (*P* < 0.05) in Control groups, but maintained unchanged in Betaine groups. As a result, the CORT-induced decrease in SAM/SAH ratio was completely restored in the liver of betaine-treated chickens (Fig. 3I).

### Plasma CORT and hepatic expression of CORT-related genes

**Figure 4:**
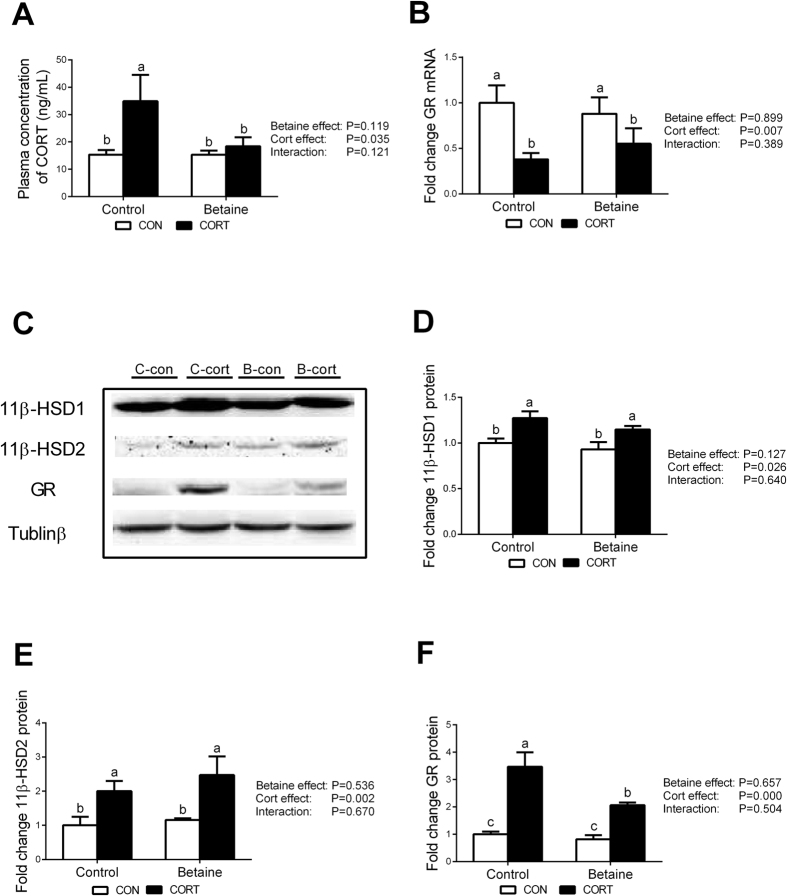
Plasma content of CORT, hepatic protein content of enzymes involved in corticosterone metabolism. (**A**) Plasma content of CORT; (**B**) Hepatic mRNA expression of GR; (**C**) Protein bands of 11β-HSD1, 11β-HSD2 and GR; (**D**) Protein expression of 11β-HSD1; (**E**) Protein expression of 11β-HSD2; (**F**) Protein expression of GR;. C = Control; B = Betaine; CON = vehicle; CORT = Corticosterone; Values are means ± SEM, means without a common letter differ, *P* < 0.05 (n = 8).

Prenatal betaine exposure did not affect plasma CORT concentration, Chronic CORT exposure increased plasma CORT concentration only in Control groups, but not in Betaine groups (Fig. 4A). Also, betaine had no effect on the hepatic expression of CORT metabolic enzymes, including 11β-HSD1 that coverts 11-dehydrocorticosterone (11DHC) to corticosterone (Fig. 4D) and 11β-HSD2 that catalyzes CORT to 11DHC (Fig. 4E). CORT injection significantly increased the protein content of these two metabolic enzymes, and betaine was not able to rescue CORT-induced up-regulation of these two CORT metabolic genes. Moreover, GR expression was not affected by betaine *in ovo* injection at either mRNA (Fig. 4B) or protein (Fig. 4F) levels. CORT challenge significantly down-regulated (*P* < 0.05) hepatic GR mRNA (Fig. 4B) yet increased (*P* < 0.05) hepatic GR protein content (Fig. 4F) in both Control and Betaine groups. However, *In ovo* injection of betaine significantly (*P* < 0.05) blunted CORT-induced increase of hepatic GR protein, but not mRNA.

### Hepatic expression of fatty acid β-oxidation and OXPHOS related genes

**Figure 5:**
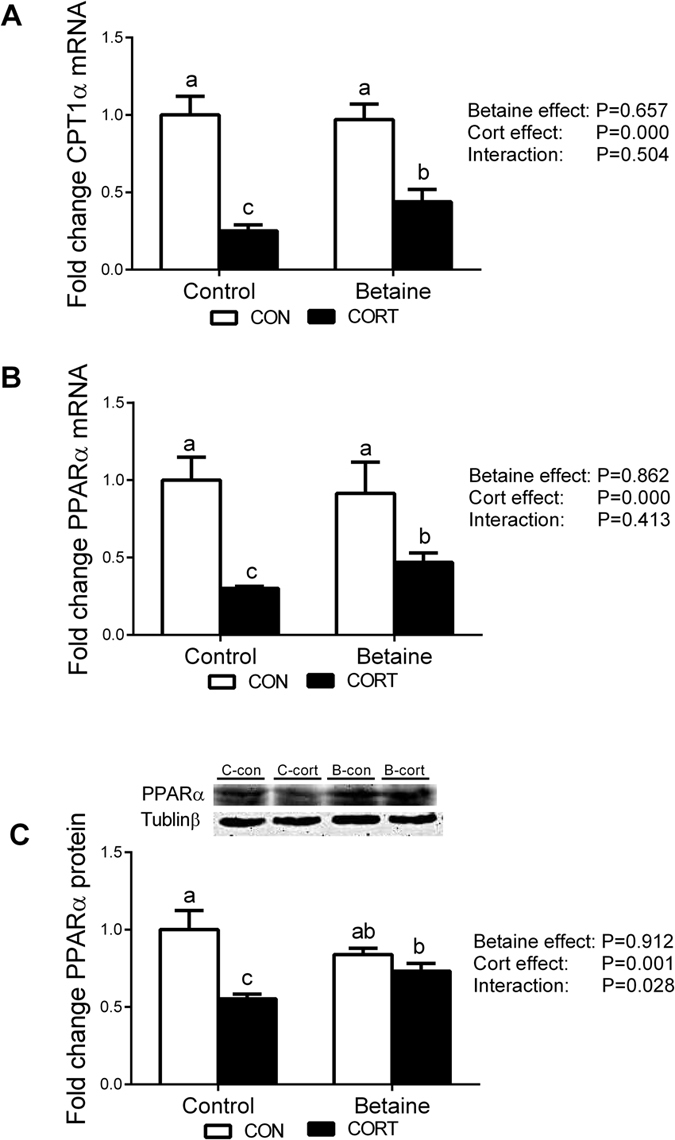
Hepatic expression of genes involved in fatty acid β-oxidation. (**A**) Hepatic mRNA expression of CPT1α; (**B**) Hepatic mRNA expression of PPARα; (**C**) Protein expression of PPARα. C = Control; B = Betaine; CON = vehicle; CORT = Corticosterone; Values are means ± SEM, means without a common letter differ, *P* < 0.05 (n = 8).

Hepatic mRNA expression of CPT1α (Fig. 5A) and PPARα (Fig. 5B) were not affected by betaine, but significantly decreased (*P* < 0.05) in response to CORT challenge in both Control and Betaine groups. CORT-induced down-regulation of CPT1α and PPARα mRNA expression was partially alleviated (*P* < 0.05) by prenatal betaine exposure. Such alleviation effect was more obvious for PPARα protein content; CORT-induced decrease (*P* < 0.05) in PPARα protein content was completely rescued in betaine-treated chickens (Fig. 5C).

### OXPHOS genes expression, mtDNA copy number and COX enzyme activity

**Figure 6:**
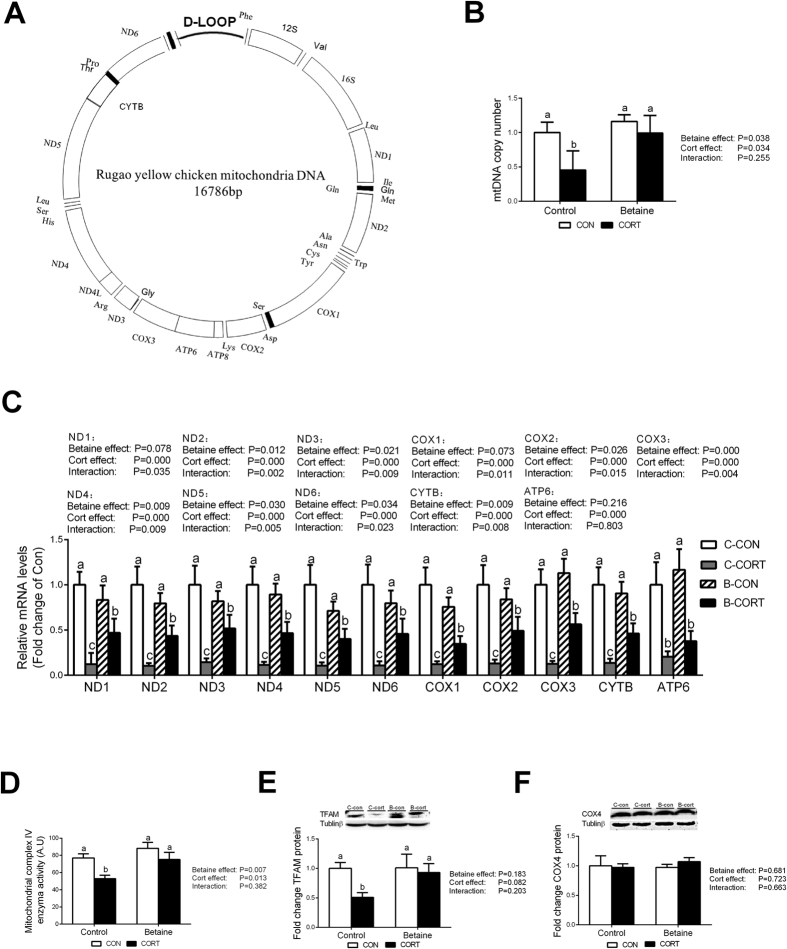
Mitochondrial and OXPHOS genes expression, mtDNA copy number and COX enzyme activity. (**A**) The structure chart of Rugao yellow chicken mitochondria; (**B**) Mitochondrial DNA copy number; (**C**) Mitochondrial genes expression; (**D**) Mitochondrial complex IV enzyme activity; (**E**) Protein expression of TFAM; (**F**) Protein expression of COX4. C = Control; B = Betaine; CON = vehicle; CORT = Corticosterone; Values are means ± SEM, means without a common letter differ, *P* < 0.05 (n = 8).

The structure chart of Rugao yellow chicken mitochondria is shown in Fig. 6A. Thirteen genes involved in OXPHOS are encoded by mtDNA. Among these 13 genes, ND4L and ATP8 sequences are too short to design specific primers for PCR analysis. Therefore, we detected the mRNA abundance of 11 mtDNA-encoded genes in this study. Chronic CORT administration significantly decreased hepatic mRNA expression of mtDNA-encoded genes (*P* < 0.05) in both Control and Betaine groups. Among 11 mtDNA-encoded genes detected, 8 genes were affected by *in ovo* betaine injection at the level of mRNA abundance (Fig. 6C). However, none of the 11 mtDNA-encoded genes were affected by betaine in ovo injection per se (B-CON vs. C-CON). Importantly, prenatal betaine exposure was able to partly alleviate CORT-induced down-regulation as shown by comparing B-CORT with C-CORT (Fig. 6C). Also, CORT administration decreased DNA copy number only in Control group (Fig. 6B). Prenatal betaine exposure did not affect DNA copy number, but prevented CORT-induced decrease (*P* < 0.05) in DNA copy number. A more obvious alleviation effect was observed for mitochondrial complex IV enzyme activity (Fig. 6D) and mitochondrial transcription factor A (TFAM) protein content (Fig. 6E); both were completely rescued in betaine-treated chickens. On the contrary, hepatic content of COX4 protein was not affected by either prenatal betaine injection or postnatal CORT challenge (Fig. 6F).

### DNA methylation status on gene promoters

**Figure 7:**
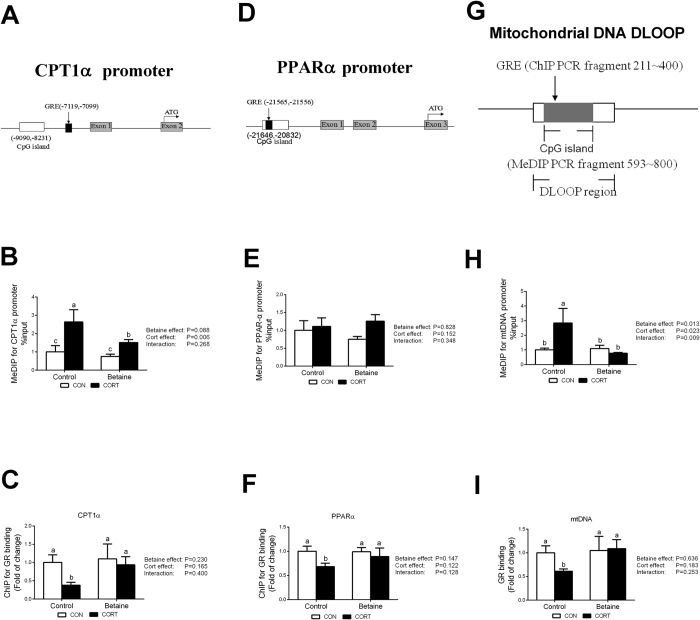
DNA methylation status and GR binding to the promoter of CPT1α and PPARα, as well as the D-loop region of mtDNA. (**A**) Schematic diagram showing the promoter sequence of chicken CPT1α gene. CpG sites (−9090~−8231) and GRE (−7119~−7110), determined in this study, on 5′-flanking promoter regions of CPT1α relative to the translation start codon (ATG) are underlined. (**B**) Methylation status on the promoter of CPT1α gene. (**C**) ChIP assays of GR binding on CPT1α gene promoter. (**D**) Schematic diagram showing the promoter sequence of chicken PPARα gene. CpG sites (−21646~−20832) and GRE (−21565~−21556), determined in this study. (**E**) Methylation status on the promoter of PPARα gene. (**F**) ChIP assays of GR binding on PPARα gene promoter. **(G**) Schematic diagram of Rugao yellow chicken mitochondrial D-loop region. The sequences of Rugao yellow chicken mitochondrial was according to PubMed (KP742951.1). CpG sites (346~905) and GRE (303~312), determined in this study. (**G**) Methylation status on the D-loop region of mtDNA. (**H**) ChIP assays of GR binding to D-loop region of mtDNA. C = Control; B = Betaine; CON = vehicle; CORT = Corticosterone; Values are means ± SEM, means without a common letter differ, *P* < 0.05 (n = 8).

CpG islands were predicted on the promoter of chicken CPT1α (Fig. 7A) and PPARα (Fig. 7D) genes, as well as the D-loop region of mtDNA (Fig. 7G). CORT administration led to significant hypomethylation on CPT1α promoter and mtDNA D-loop region. *In ovo* injection of betaine did not affect the level of methylation on CPT1α promoter (Fig. 7B) or mtDNA D-loop region (Fig. 7H), but was able to alleviate CORT-induced hypermethylation (*P* < 0.05) on these regulatory sequences. Niether betaine nor CORT caused significant alteration in the methylation of PPARα promoter (Fig. 7E).

### GR binding to the promoter of affected genes

ChIP was employed to analyze the GR binding to the promoter sequences of CPT1α (Fig. 7A) and PPARα (Fig. 7D) genes, as well as the D-loop region of mtDNA (Fig. 7G) which are predicted to contain glucocorticoid receptor element (GRE) binding sites. CORT administration decreased GR binding to CPT1α (Fig. 7C), PPARα (Fig. 7F) gene promoter regions and mtDNA D-loop region (Fig. 7I) only in Control group. Prenatal betaine exposure did not affect GR binding to CPT1α, PPARα gene promoter regions, or mtDNA D-loop region, but was able to completely alleviate CORT-induced down-regulation (*P* < 0.05) of GR binding to these regulatory regions.

## Discussion

In this study, we provide the first evidence that *in ovo* injection of betaine promotes posthatch growth and alleviate CORT-induced hepatic lipid accumulation in the chicken. The growth promoting effect of betaine observed in the present study was in agreement with the previous reports in neonatal piglets born to betaine-supplemented sows^36^. The mechanisms underlying the growth promoting effects of betaine involve stimulation of growth hormone (GH) secretion and autocrine/endocrine IGF-1 release^23^,^37^. Yet, it remains to be elucidated whether GH/IGF-1 system is programmed by *in ovo* betaine administration in the chicken.

The liver protection effect of betaine observed in the present study is in line with the previous reports that betaine protects against high-fat-diet-induced liver injury in rats^30^,^31^,^38^,^39^ and alleviates carbon tetrachloride-induced liver injury in chickens^40^. Recently, it is reported that dietary supplementation of betaine to sows during gestation significantly decreases the hepatic lipid deposition in neonatal piglets^32^, thus indicating a programming effect of maternal betaine on offspring lipid metabolism in the liver. However, the pig study describes only the alteration of hepatic lipid content under basal situation and it remains elusive whether betaine-induced decrease of hepatic lipid deposition in the neonatal stage would affect the susceptibility of fatty liver in later life. In this study, the juvenile chickens, with or without prenatal betaine exposure, were challenged by repeated CORT injection for 7 days to establish a model of stress-induced fatty liver. The protective effect of *in ovo* betaine administration on fatty liver syndrome is indicated by the gross appearance of the liver, the oil-red staining of the sections, and the biochemical quantification of hepatic triglycerides contents.

Prenatal betaine exposure was reported to modulate lipogenic gene expression in the liver of neonatal piglets^32^. In this study, we found that prenatal betaine treatment was able to prevent CORT-induced down-regulation of genes involved in fatty acids β-oxidation, such as *PPARα* and *CPT1α*. This is in line with previous reports that betaine alleviates hepatic lipid accumulation via enhancing fatty acid oxidation, with activated expression of *PPARα* and *CPT1α* genes^30^,^41^. Our findings together with others indicate that enhanced hepatic β-oxidation of fatty acids contributes, largely, to the liver protection effects of betaine. This presumption is further supported by the expression of mtDNA-encoded OXPHOS genes. Mitochondria is the main organelle of fatty acids β-oxidation and plays an active role in many metabolic pathways including lipid homeostasis^42^,^43^. Betaine was reported to increase the mitochondrial membrane potential in mouse hepatic cells *in vitro*^44^. Furthermore, betaine was shown to attenuate the liver injury and oxidative stress induced by ethanol through improving mitochondrial function in rats^45^. In this study, *in ovo* injection of betaine significantly rescued the CORT-induced down-regulation of mtDNA copy number, mtDNA-encoded genes expression and mitochondrial complex IV activity. TFAM, a key activator of mtDNA replication and transcription^46^, may be involved in the regulation.

Previously we reported that *in ovo* injection of betaine induces epigenetic modification of cholesterol metabolic genes in the liver of newly hatched chicks^33^. Also, epigenetic changes of hepatic mtDNA was observed in NAFLD patients^47^. Therefore, we hypothesized that *PPARα* and *CPT1α* genes, as well as the D-loop region of mtDNA may subject to epigenetic regulation in betaine-exposed chickens. However, *in ovo* injection of betaine had no effect on the methylation of PPARα, CPT1α gene promoter sequences and D-loop region of mtDNA under basal situation. Under CORT challenge, both the promoter sequence of CPT1α gene and the D-loop region of mtDNA were hypermethylated, which was associated to decreased mRNA abundance of these genes. Interestingly, all these CORT-induced alterations in DNA methylation were vanished in betaine-treated chickens. The mechanism underlying the protective effects of prenatal betaine on CORT-induced postnatal epigenetic modifications remains a mystery. However, one-carbon metabolic pathway and DNA methyltransferases (DNMTs)^48^ appear to play a role. DNMT1 is the only member of the three known catalytically active DNA methyltransferases that targets to the mitochondrion^49^. The protein content of DNMT1 in the liver was increased upon CORT injection in control chickens but not betaine-treated chickens. Also, the CORT-induced decrease in SAM/SAH ratio in the liver was completely rescued in betaine-treated chickens. Nevertheless, the transcriptional regulation of gene expression is complex and mismatches between the methylation level of the promoter and the mRNA abundance of the gene were also observed. For instance, no significant alteration was detected for the methylation of PPARα promoter, whereas its mRNA expression was significantly down-regulated in CORT-treated chickens.

Majority of the corticosterone biological functions are mediated through GR activation. GR is a transcription factor that binds to its GRE in the regulatory region of their target genes. Our previous publication reported that betaine supplementation during gestation led to diminished GR binding on the promoter of porcine SCD gene in the liver of neonatal piglets^32^. The chicken *PPARα* and *CPT1α*, as well as mtDNA-encoded genes, are predicted to have GR binding sites (GREs) on the promoter, or the D-loop region of mtDNA. In accordance with the CORT-induced down-regulation of these genes, GR binding to the regulatory regions of these genes was also diminished in CORT-challenged chickens. Again, CORT-induced decrease in GR binding was observed only in control but not betaine-treated chickens. These results indicate that prenatal betaine exposure can modulate the corticosterone signaling in response to stress or CORT challenge. Indeed, *in ovo* betaine administration completely abolished the response of plasma corticosterone level to exogenous CORT challenge. Upon CORT challenge, plasma corticosterone level was elevated only in control but not betaine-treated chickens. Several factors may contribute to such difference, which includes adrenal steroidogenesis, corticosterone release and clearance. Also, the changes of GR mRNA abundance, GR protein content, and GR binding to target gene promoters in response to CORT-challenge appear to contradict one another. For instance, GR mRNA was down-regulated, while total GR protein was increased, and GR binding to target genes (PPARα, CPT1α, and mtDNA-encoded genes) was diminished, in the liver of CORT-challenged control chickens. These contradictory results implicate complex regulation of GR expression, activation and function. Future in-depth studies are needed to unravel the mechanisms of post-transcriptional and translational regulation, as well as mitochondria and nuclear translocation and function of GR in the chicken especially under stress situation, such as CORT challenge in this study.

In conclusion, we demonstrate, for the first time, that *in ovo* injection of betaine allivates CORT-induced fatty liver in the chicken through epigenetic and GR-mediated regulation of mitochondrial β-oxidation and OXPHOS genes. Obviously, several questions are not resolved in the present study which may provide directions for future investigation. Firstly, an in-depth mechanistic study is required to elaborate the effect of betaine on corticosterone biosynthesis, release and clearance. Secondly, dietary betaine supplementation to laying hens, instead of *in ovo* betaine injection, should be performed to evaluate whether dietary supplementation may increase betaine deposition in the breeder eggs, and whether higher betaine content in the egg can prevent stress- or CORT-induced fatty liver disease hatched chicks later in life. Finally, rodents or *in vitro* cell models may be used to understand whether the fetal programming of liver protection effects of betaine, as observed in the chicken models, can be extrapolated to mammalian species and even human clinical trials.

## Materials and Methods

### Ethics Statement

The experimental protocol was approved by the Animal Ethics Committee of Nanjing Agricultural University, with the project number 2012CB124703. Animal care and handling are in compliance with regulations of AVMA Guidelines for the Euthanasia of Animals: 2013 Edition. The sampling procedures complied with the “Guidelines on Ethical Treatment of Experimental Animals” (2006) No. 398 set by the Ministry of Science and Technology, China.

### Animals and treatment

Fertilized eggs (42.46 ± 0.21 g, ranging from 39.00 g to 44.42 g) laid by Rugao yellow breeder hens were obtained from Poultry Institute of Yangzhou, Jiangsu, China. Before incubation, eggs were randomly divided into control (C) and betaine (B) groups (230 in each group), and were injected with 100 μL of saline or betaine (2.5 mg per egg, B2629, Sigma–Aldrich, USA), respectively, as previously described^33^. In brief, eggs were injected by advancing a Hamilton syringe into a hole in the middle of the long axis until the yolk membrane was penetrated (approximately 20 mm below the surface). Chicks were hatched inside the incubator and were left to dry completely (up to 12 h) before they were removed. No obvious differences in hatchability or hatching time were observed between two groups. One-day-old chicks were individually weighed, wing labeled and raised according to the standard recommended by the breeder. Chicks were subjected to continuous illumination and the temperature was controlled in the range of 35–37 °C during the first week, and reduced approximately 3 °C per week until 21 °C. The breeding density was about 30 kg/m^2^. The relative humidity was maintained at 40–60%, and the lighting, ventilation, as well as the feeding and management procedures complied with the Feeding Management Regulations of Ru-gao Chickens. At 8 weeks of age, 24 male chickens were selected from both C and B groups and chickens in each group were divided to two subgroups, being subjected to either vehicle (CON) or corticosterone (CORT) treatment with daily subcutaneous injection of solvent (15% ethanol) or corticosterone (C2505, Sigma–Aldrich, USA) in a dose of 4.0 mg/kg body mass for 7 days (twice per day, 9:00–10:30 and 21:00–22:30). Growth performance was recorded weekly from hatching to 9 weeks of age. At 64 days of age, all the chickens in 4 groups (12 chickens in each group), C-CON, C-CORT, B-CON and B-CORT, were weighed and killed by rapid decapitation which is considered to be acceptable for euthanasia of birds according to American Veterinary Medical Association (AVMA) Guidelines for the Euthanasia of Animals: 2013 Edition. Blood samples were taken and plasma samples were separated and stored at −20 °C. Liver (without the gall bladder) samples were rapidly frozen in liquid nitrogen and keep at −80 °C for further analysis.

### Determination of hepatic content of triglyceride and plasma concentration of triglyceride and corticosterone

Triglyceride in plasma and liver was measured by using respectively commercial triglyceride assay kit (E1003 and E1013) purchased from Applygen Technologies Inc., China, following the manufacturer’s instructions. Plasma CORT concentration was measured using a commercial EIA kit (No. ADI-900–097, Enzo, USA) according to the instructions of the manufacturer.

### Total RNA isolation and real-time PCR

Total RNA was isolated from liver sample (30 mg) using TRIzol Reagent (Invitrogen, USA) and then treated with RNase-free DNase and reverse-transcribed to cDNA using random hexamer primers (Promega, USA). Two microliters of diluted cDNA (1:20, vol/vol) was used for real-time PCR with a Mx3000 P Real-Time PCR System (Stratagene, USA). All primers (Table 1) were synthesized by Generay Biotech (Shanghai, China). Several references genes were tested and 18 S rRNA that is not affected by the experimental factors (betaine & CORT) was chosen as a reference gene. Data were analyzed using the method of 2^−ΔΔCT50^.

### Total protein extraction and western blotting

Total protein was extracted from 80 mg frozen liver sample as previously described^51^. The protein concentration was determined according to the manufacturer’s instructions of Pierce BCA Protein Assay kit (Rockford, IL, USA). Forty or sixty micrograms of protein were used for electrophoresis on a 7.5% or 10% SDS-PAGE gel. Western blot analysis for methionine adenosyltransferase 2B (MAT2B) (15952–1-AP, Proteintech, USA, diluted 1:1000), DNA (cytosine-5-)-methyltransferase 1 (DNMT1) (sc-20701, Santa Cruz, UK, diluted 1:200), GR (sc-1004, Santa Cruz, UK, diluted 1:200), 11β-HSD type 1 (11β-HSD1) (ab109554, Abcam, UK, diluted 1:1000), Type 2 11β-HSD (11β-HSD2) (sc-20176, Santa Cruz, UK, diluted 1:200), PPARα (ab2779, Abcam, UK, diluted 1:1000), mitochondrial transcription factor A (TFAM) (BS7319, Bioworld Technology, USA, diluted 1:1000), COX4 (AP0707, Bioworld Technology, USA, diluted 1:1000) were carried out according to the recommended protocols provided by the manufacturers. Images were captured by VersaDoc 4000MP system (Bio-Rad, USA) and the band density was analyzed with Quantity One software (Bio-Rad, USA).

### Determination of mtDNA copy number and COX enzyme activity

High-quality genomic DNA was isolated from liver tissues and the mtDNA copy number was determined using real-time PCR as previously described^52^. The specific primers of 18 S rRNA were used for standardization (Table 1). Relative mtDNA copy number was calculated with the method of 2^−ΔΔCT^.

For measuring COX enzyme activity, liver mitochondria were isolated according to a previously described protocol^53^. Briefly, 100 mg of frozen liver samples (w:v =1:5) were homogenized in an isolation buffer (0.1 M Tris-MOPS, 0.1 M EGTA/Tris, and 1 M sucrose) with a Dounce homogenizer. Then the homogenates were centrifuged at 600 g, 4 °C for 10 min. The supernatant was collected and centrifuged at 7000 g, 4 °C for 10 min. The supernatant was removed and the precipitate was resuspended in the isolation buffer. Mitochondrial complex II (GMS50008) and IV (GMS500010) activities were determined according to the instruction of respective commercial kits (Genmed Scientifics, Inc. Shanghai, China).

### Methylated DNA immunoprecipitation (MeDIP) analysis

Genomic DNA was isolated from the liver tissues and sonicated to produce small fragments ranging from 300 to 1000 bp. The fragmented DNA (2 μg) was heat-denatured to produce single-stranded DNA, and the immunoprecipitation was performed overnight at 4 °C with 2 μg antibody against 5-methyl cytosine (ab10805, Abcam, UK). Pretreated protein A/G agarose beads (40 μL, 50% slurry, sc-2003, Santa Cruz, USA) were used to capture the antibody/DNA complexes. The beads bound to immune complexes were washed to remove nonspecific binding and resuspended in 250 μL digestion buffer containing proteinase K. Finally, the MeDIP DNA was purified. A small aliquot of MeDIP DNA and control input DNA was used to amplify the proximal promoter sequence of chicken GR, CPT1α and PPARα genes, as well as the D-loop region of mtDNA by real-time PCR with specific primers listed in Table 1. Data was normalized against the input and presented as the fold change relative to the average value of the C-CON group.

### Chromatin immunoprecipitation (ChIP) assay

ChIP analysis was performed according to our previous publications with some modifications^17^. Briefly, 300 mg of frozen liver tissues were ground in liquid nitrogen, resuspended in PBS containing protease inhibitor cocktail (no.11697498001, Roche, USA) and cross-linked in 1% formaldehyde for 10 min, then the reaction was stopped with 2.5 M glycine at room temperature for 10 min. The pellets were washed with PBS and homogenized with SDS lysis buffer containing protease inhibitors cocktail. Chromatin samples were sonicated to produce small fragments ranging from 300 to 1000 bp and precleared with salmon sperm DNA-protein A/G agarose (40 μL, 50% slurry). The mixture of precleared chromatin was incubated with 2 μg specific GR antibody overnight at 4 °C (sc-1002x, Santa Cruz). Normal IgG (12–370, Millipore) was used as negative control. Protein A/G agarose beads (40 μL, 50% slurry) were added to capture the immunoprecipitated chromatin complexes. Finally, DNA fragments was released from the immunoprecipitated complex by reverse cross-linking at 65 °C for 5 h, and the immunoprecipitated DNA was purified. The immunoprecipitated DNA was used as template for real-time PCR and to quantify the fragments of chicken GR, CPT1α, PPARα promoter, as well as the D-loop region of mtDNA with specific primers listed in Table 1.

### Statistical analysis

Descriptive statistics was performed to check the normality and homogeneity of variances before using parametric analyses. Log^10^ transformation was performed before statistical analysis when the data distribution was not normal. Body weight was analyzed by repeated measures in the General Linear Model (GLM) procedure. For other parameters involving 4 groups, two-way ANOVA was performed to assess the main effects of Betaine and CORT, as well as their interactions using the General Linear Model, followed by LSD post hoc analysis to evaluate differences between specific groups. All analyses were performed using SPSS 20.0 software (SPSS Inc., Chicago, IL, USA). Data are expressed as means ± SEM. The differences were considered statistically significant when P < 0.05.

## Additional Information

**How to cite this article**: Hu, Y. *et al*. In ovo injection of betaine alleviates corticosterone-induced fatty liver in chickens through epigenetic modifications. *Sci. Rep.* **7**, 40251; doi: 10.1038/srep40251 (2017).

**Publisher's note:** Springer Nature remains neutral with regard to jurisdictional claims in published maps and institutional affiliations.

## Figures and Tables

**Table 1:** Nucleotide sequences of specific primers.

Target genes	Primer sequences (5′ to 3′)	Used for
PPARα	F: AGGAAATCTACAGGGACA	mRNA quantification
	R: GAACCGAGTGAACAGC	
CPT1α	F: GGCATTGACCGCCATCTGT	mRNA quantification
	R: GAAACACCGTAACCATCATCAGC	
GR	F: CTTCCATCCGCCCTTCA	mRNA quantification
	R: TCGCATCTGTTTCACCC	
COX1	F: CTACTAGCCTCATCTACCG	mRNA quantification
	R: TAGAATGGAGGAAACACC	
COX2	F: CACGCCCTGATAGTCGC	mRNA quantification
	R: CTACGGTGTTTGATGATAGTTT	
COX3	F: ACACCCCAACTGTCCAAAAG	mRNA quantification
	R: TGCGTGGATGGCTTGTT	
ND1	F: GCATGACCCCTCGCAATA	mRNA quantification
	R: GGAATAGGACGGTGGTTAGTGT	
ND2	F: CCTAATCGGAGGCTGAATG	mRNA quantification
	R: GGTGAGAATAGTGAGTTGTGGG	
ND3	F: TAACCCTTACTTGAGCCACCAC	mRNA quantification
	R: GCCCTGAGTTCATTCGTAGA	
ND4	F: CCAACCACCAACCTGATAGC	mRNA quantification
	R: TGTGGGATGGAAGAGTGCC	
ND5	F: GGATGATGACAAGGACGAGC	mRNA quantification
	R: TGGGTGTTTGGTTTGGGT	
ND6	F: CCCCGCCATCAACAGTAA	mRNA quantification
	R: GTGGTAGCGTCTGTGATAGGAT	
ATP6	F: TAATCCTCCCATCACTCC	mRNA quantification
	R: TTTGTGACCTGCCTTGT	
CYTB	F: ACTATCATCCACCTCACCT	mRNA quantification
	R: AGTACACCTCCAAGTTTGTT	
18 S rRNA	F: ATAACGAACGAGACTCTGGCA	mRNA quantification
	R: CGGACATCTAAGGGCATCACA	
GR	F: GCGTGGGAAGTTCGGATAAG	MeDIP
	R:TCACCGTCGTTCTGTAAATAAGC	
PPARα	F: CGTTGGGCAGTTTGGC	MeDIP
	R:TAGCGTGAGGTACTAACAGGAA	
CPT1α	F: GTTAGGCAGGCGAAGCAC	MeDIP
	R:ACCGCAGTCGGGAAGTAG	
mtDNA	F:TGGATTATCTTCCCCTCTT	MeDIP
	R:TCCCCATACACGCAAA	
mtDNA	F: AAGACATATTCATTCACCCTC	ChIP
	R:TCTGATACGGCGAGCA	
PPARα	F: TGGCAGCGAGCGGAAAA	ChIP
	R:GCACTCCCTCCCGTGACTC	
CPT1α	F: AGCCCTCAGAAATAGC	ChIP
	R:TTTGATTCACGCACAG	
